# Cancer screening in Koreans: a focus group approach

**DOI:** 10.1186/s12889-018-5147-9

**Published:** 2018-02-14

**Authors:** Shin-Young Lee, Eunice E. Lee

**Affiliations:** 1 Department of Nursing, Chosun University, 309 Pilmun-daero, Dong-gu, Gwangju, 501-759 Republic of Korea; 2 School of Nursing, University of California, Los Angeles, CA USA

**Keywords:** Cancer, Screening, Koreans

## Abstract

**Background:**

Cancer is the greatest disease burden in Korea. Cancer screening can reduce the burden of cancer but cancer screening rates among Koreans remain low. The purposes of this study were to a) understand Koreans’ beliefs and knowledge about cancer screening, and b) explore preferred strategies for increasing cancer screening utilization.

**Methods:**

We conducted a descriptive, qualitative study using eight face-to-face focus groups with a total of 64 Koreans aged 40 and over. Participants answered semi-structured, open-ended questions assessing their experiences with, and beliefs, knowledge, and opinions about, cancer screening. All interview data were recorded and analyzed in the context of the health belief model (HBM).

**Results:**

The most important themes that emerged from the focus group data were (a) perceived susceptibility (most of the participants believed they were not susceptible to cancer; those who perceived themselves susceptible to cancer were reluctant to express it); (b) perceived benefits (early detection and feelings of relief after cancer screening were benefits; participants had screening because they wanted to take advantage of the Korean government’s Medical Payment Support program for cancer patients who have participated in the National Cancer Screening program); (c) perceived barriers (no symptoms; self-care when having symptoms; widespread distrust of tests, doctors, and hospitals; unkind health care providers; the financial burdens of advanced cancer screening tests; and the discomfort during cancer screening); and (d) knowledge of the causes of cancer (incorrect knowledge including beliefs that stress, personality, and body overuse cause cancer). Almost all of the participants were very knowledgeable about the seriousness of cancer and were confident that they were able to have cancer screening. Participants preferred strategies of cancer screening using group interventions with family or friends; various information delivery methods; information emphasizing the importance of cancer prevention; convenient, free, or inexpensive services; and kind health care providers.

**Conclusions:**

This HBM-based research suggests that beliefs in low susceptibility to cancer, many barriers to cancer screening, and incorrect knowledge should be the foci for increasing cancer screening rates in Koreans. Interventions could change individual cultural beliefs and increase knowledge as well as the quality of health care for Koreans.

## Background

Cancer is the greatest disease burden in Korea. The most recent data show that in 2014, 217,057 Koreans were diagnosed with cancer; the incidence was 312.4 per 100,000 men and 282.9 per 100,000 women in Korea [1]. In 2014, 77,902 Koreans died of cancer, which accounted for 29.1% of all deaths; mortality was 157.5 per 100,000 men and 70.4 per 100,000 women [2].

In an effort to reduce the burden of cancer, the Korean National Health Insurance Service has provided National Health Insurance beneficiaries and Medical Aid recipients with screening for five types of cancer (breast, cervical, gastric, colorectal, and liver) at low cost or free through the National Cancer Screening Program since 2005 [3]. This program includes the following tests: (a) breast cancer screening with mammograms every 2 years for women aged 40 and older, (b) cervical cancer screening using Pap smears every 2 years for women aged 20 and older, (c) gastric cancer screening using an upper gastrointestinal series or upper endoscopy every 2 years for both men and women aged 40 and older, (d) colorectal cancer screening using an occult fecal blood test every year for both men and women aged 50 and older, and (e) liver cancer screening using hepatic ultrasonography and measuring alpha-fetoprotein levels every 6 months for high-risk groups (positive for hepatitis B virus surface antigen, hepatitis C virus antibodies, or presence of liver cirrhosis) aged 40 and older [3]. In addition to these screening tests provided by the National Health Insurance Service, individuals can choose many other cancer screening options if they are willing to pay for the service. Even so, cancer screening rates in Korea are suboptimal. For instance, recent data on cancer screening rates in 2012 among Korean men aged 40 years and older and women aged 30 years and older show that the lifetime screening (ever having had screening) rates for breast, cervical, gastric, colorectal, and liver cancers were 82.9%, 77.1%, 77.9%, 65.8%, and 69.9%, respectively [4]; and the rates for breast, cervical, gastric, colorectal, and liver cancers screening according to the recommended guidelines were 70.9%, 67.9%, 70.9%, 44.7%, and 21.5%, respectively [4].

The literature shows that cancer screening utilization in Korea is associated with socio-demographic factors (age, gender, and residential area), cognitive factors (knowledge, health, and cultural beliefs), and health care system factors (e.g., the National Cancer Screening Program) [5–16]. For example, Lim and Kim [12] analyzed the Korea National Health and Nutrition Examination Survey with 2928 Koreans aged 50 and older and identified that socio-demographic factors such as age, gender, and residential region were significantly different between the colorectal cancer screening group (having had colonoscopy, double-contrast barium enema, flexible sigmoidoscopy, or FOBT during the previous 5 years) and the non-colorectal cancer screening group. In that study [12], Koreans aged 70 and older (22.0%) were less likely to undergo colorectal cancer screening than those aged 50 to 59 (35.5%) or 60 to 69 (36.6%). Also, more men (36.1%) than women (29.1%) participated in colorectal cancer screening, and urban residency (33.7%) was significantly associated with higher rates of colorectal cancer screening compared to rural residency (29.3%). Associations between demographic variables (age, gender, and residency) and cancer screening have been identified [9, 13–15], but there is little research on why and how Koreans’ cancer screening behaviors differ by age, gender, and residency. A qualitative study using focus groups found that Koreans’ cultural beliefs (e.g., Korean women’s discomfort with male physicians) and issues related to the health care system (e.g., dissatisfaction) were barriers to cervical cancer screening procedures such as the Pap test [10]. Another focus group study revealed that one of barriers to cancer screening for Medical Aid Program recipients was lack of trust in the National Cancer Screening program participants because they believed that the free screening services were low quality [16]. These findings indicate that cultural beliefs and health care system issues need to be deeply explored to improve cancer screening utilization among Koreans.

Given that:

1. Tremendous governmental efforts such as the National Cancer Screening program as well as non-governmental programs have been ongoing for decades, but cancer screening rates among Koreans remain low.
2. Most of the studies on cancer screening have used quantitative research designs with only a few qualitative ones [10, 11, 16], and this means that there is a paucity of recent in-depth information about Koreans’ beliefs, knowledge, and preferences concerning cancer screening.

Therefore:

These beliefs and preferences and this knowledge should be explored in depth in order to design the most effective messages and interventions to better motivate participation and thus raise cancer screening rates.

Strategies for increasing participation in cancer screening programs are essential, and efficacious interventions should generate a higher degree of motivation and participation [17]. In-depth data on Koreans’ preferences for cancer screening strategies can provide a blueprint for designing the most effective messages and interventions to better motivate participation.

This study used the health belief model (HBM) that has been a notable explanatory and predictive model in health behavior research and interventions in order to understand health-related factors influencing cancer screening [18, 19]. It was initially developed by psychologists in the U.S. Public Health Service in order to understand the low participation of individuals in programs to prevent and detect diseases [20]. Core concepts of the HBM include perceived susceptibility, perceived severity, perceived benefits, perceived barriers, self-efficacy, and cues to action [18]. From the HBM perspective, people will comply with cancer screening if they consider themselves to be susceptible to cancer; if they believe that cancer has serious consequences, that cancer screening would be beneficial, and that barriers to cancer screening are outweighed by its benefits; and if they are confident in their ability to access cancer screening. When beliefs allow it, cues to action can be effectively applied by providing education; promoting awareness; and employing a reminder system, mass media campaigns, and advice from health care providers [18]. These cues can provide the basis for an effective prevention program. Other factors such as age, ethnicity, education, socioeconomic status, and knowledge of health issues also affect preventive behaviors [18]. In the context of the HBM, this study attempted to clarify the factors involved in cancer screening in order to develop effective prevention programs and increase cancer screening utilization.

The purposes of this study were to a) understand Koreans’ beliefs and knowledge about cancer screening in-depth and b) explore preferred strategies for improving cancer screening utilization. This study will contribute to the understanding of what Koreans believe and know about cancer screening in the health care systems and of why cancer screening rates among Koreans are still suboptimal despite the National Cancer Screening program. It will provide feasible and culturally appropriate interventions to increase cancer screening utilization and will eventually decrease cancer incidence and cancer-related mortality rates.

## Methods

### Study design

A descriptive, qualitative approach using face-to-face focus group interviews was used in this study. Qualitative research provides a greater depth of information by exploring beliefs and knowledge about, and preferred strategies for, cancer screening in Korea. Focus groups were chosen over individual qualitative interviews because group discussions are more dynamic and require less time and resources to conduct [21]. The focus group method was also useful in eliciting from study participants their preferred strategies for increasing cancer screening.

### Participants

Koreans who (a) resided in Korea, (b) were age 40 and older, and (c) had no history of cancer and no first-degree relatives with cancer were included in this study. The reasons for the selection of this population were twofold: The National Cancer Screening program provides cancer screening for Koreans focusing on middle-aged and older citizens [3], and people with a history of cancer or of first-degree relatives with cancer may have different beliefs and knowledge about cancer and cancer screening than people who do not.

Koreans were recruited using convenience sampling from community centers, senior centers, and churches in an urban city and rural areas to find eligible participants. Rural and urban males and females in the 40 to 64 and 65 and over age groups were recruited to explore gender, age, and regional differences in beliefs and knowledge about cancer screening. Recruitment continued as long as new themes continued to emerge from the interviews, and data saturation was reached at a sample of eight focus groups with 64 participants [22]. No refusals from any of the individuals in the groups were reported.

### Data collection

Eight focus groups, each consisting of 5–9 Koreans, were held at community centers, senior centers, and churches. We developed focus group questions following the model of Ruff and colleagues [23], using open-ended questions that included introductory questions, key questions, and a concluding question. Introductory questions such as, “How do you view cancer screening?” encouraged people to respond from their own set of beliefs and experiences. Key questions focused on cancer screening experiences, beliefs, knowledge, and preferred strategies, with questions such as, “Have you had a cancer screening test?” “What would you say about your experience with a test?” “How likely is it that you will get cancer?” “What happens if a person has cancer?” “What are the benefits of and barriers to getting cancer screening tests?” “How confident are you that you could get a cancer screening test if you wanted to?” and “What do you think are the most preferred and effective strategies for a program to improve cancer screening utilization?” We asked concluding questions to ensure that complete information had been obtained. At the end, we asked, “Is there anything else you would like to say about cancer screening?” to give participants an opportunity to further elaborate on any of their thoughts or experiences.

The principal investigator (PI) moderated the focus group interviews in Korean using a semi-structured, open-ended focus group interview guide, while the second author took notes on the participants’ comments as the interviews were being recorded. After signing a consent form, participants were questioned about cancer and cancer screening. Information from each group was validated by other groups during interviews. Each focus group interview lasted approximately 60 to 90 min. After the interview, participants were asked to fill out a brief background questionnaire about their age, gender, marital status, education, income level, health insurance, and history of cancer screening utilization. Cancer screening history was measured by asking about breast, cervical, gastric, and colorectal cancer screenings during an individual’s lifetime and the time of the most recent screening.

### Data analysis

Six research assistants who were nursing students transcribed the focus group interviews verbatim. Transcribed texts were coded using standard, descriptive, and qualitative content analysis [24]. Three researchers who had doctoral degrees in health-related areas and who were experts in Korean culture and cancer screening research independently coded participants’ responses. They used inductive coding to capture all content and to categorize the coded responses as beliefs and knowledge about, and preferred strategies for, cancer screening. Researchers conducted coding reflecting maximized descriptions of the phenomena and avoiding interpretation of the phenomena [25]. Phrases and words that the participants frequently mentioned during focus group interviews were used as themes [26]. The coding and categorization of each response were not finalized until coders reached a consensus by researchers. Descriptive statistics were calculated for socio-demographic information and cancer screening utilization of the participants.

### Rigor of the study

The accuracy of the findings was validated using several strategies. Cross-member checking [27] by asking successive participants about the findings, including emergent themes, was used to ensure the accuracy of the findings for the duration of the focus group interviews. Researchers kept journals recording their reflections including self-critiques and self-appraisals [28]. All critical information about the literature reviews, the content of the interviews, and the interpretations of the researchers were described in order to critique and evaluate each researcher’s own values and biases. We also examined and triangulated different data sources of information such as observational field notes, contents of the interviews, and the researchers’ personal reflective journals and used them to build coherent justifications for the themes.

### Ethical considerations

The Institutional Review Board of the university approved the research protocol. Before consent forms were signed, written information was given to the participants about the purpose and procedures of the study, protection of privacy and confidentiality, and the PI’s contact numbers. The PI explained that participants could refuse to answer any questions and could withdraw from the focus group at any time if they were uncomfortable. Information obtained in connection with this study was kept confidential throughout the process.

## Results

### Sample characteristics

A total of eight focus groups were conducted, characterized by age, sex, and residency (see Table 1): four middle-aged (40–64 years old) and four older (65 years or more) groups; two male and six female groups; and five urban and three rural groups. General characteristics of the participants in the focus groups are displayed in Table 2. Cancer screening history was examined by having ever had a cancer screening and having cancer screening with recommendations according to the guidelines.

**Table 1:** Focus groups characteristics (*N* = 8)

Group number	Age	Gender	Residential area
1	40–64	Female	Urban
2	40–64	Female	Rural
3	40–64	Female	Urban
4	40–64	Male	Urban
5	65 and older	Female	Urban
6	65 and older	Female	Urban
7	65 and older	Male	Rural
8	65 and older	Female	Rural

**Table 2:** General characteristics of participants (*N* = 64)

	*N*	(%)	*M*	*SD*	Range
Age			63.73	11.84	41–83
40–64	32	(50.0)			
65 and older	32	(50.0)			
Gender					
Male	13	(20.3)			
Female	51	(79.7)			
Marital Status					
Currently married	47	(73.4)			
Widowed	17	(26.6)			
Education					
High school diploma or less	54	(84.4)			
Higher than high school diploma	10	(15.6)			
Employment					
Full-time	32	(50.0)			
Part-time	2	(3.1)			
Not employed	30	(46.9)			
Health Insurance					
National Health Insurance	62	(96.9)			
Medical Aid program	2	(3.1)			
Annual household income					
≤ $30,000	48	(75.0)			
> $30,000	16	(25.0)			
Cancer Screening Experience					
Breast cancer screening (*N* = 51)					
Ever had a mammogram	45	(88.2)			
Had a mammogram in previous 2 years	39	(76.5)			
Cervical cancer screening (*N* = 51)					
Ever had a Pap smear test	47	(92.2)			
Had a Pap smear test in previous year	35	(68.6)			
Gastric cancer screening					
Ever had gastric endoscopy	45	(70.3)			
Had gastric endoscopy in previous 2 years	31	(48.4)			
Colorectal cancer screening					
Ever had FOBT	53	(82.8)			
Had FOBT in previous 1 year	29	(45.3)			
Ever had a colonoscopy	30	(46.9)			
Had a colonoscopy in previous 10 years	24	(37.5)			

### Beliefs and knowledge about cancer and cancer screening

According to the content analysis of the data, beliefs that participants mentioned during the interview were categorized by core concepts of the HBM, including perceived susceptibility, perceived severity, perceived benefits, perceived barriers, self-efficacy, and knowledge (see Table 3). Regarding perceived susceptibility to cancer, we found two themes: robustness and anxiety. Most of participants in the focus groups believed that they were healthy and would not get cancer, while some expressed anxiety about having cancer someday. Interestingly, although the latter group thought they were susceptible to cancer, they did not appear to express those feelings. One middle-aged man said,

I cannot express it, even though I believe that I might have cancer someday. I feel that way, but I cannot say so. As the Korean proverb says, “Words have a way of coming true,” which means you should be careful what you say. It may come true. Therefore, I cannot say if I am susceptible to cancer. I try to believe that I will not have cancer. However, I have some anxious feelings. I insured myself because I have a feeling that I may get the disease. People may think that they might be sick due to cancer but they will not express those feelings. That is how people behave.

**Table 3:** Beliefs and knowledge about cancer and cancer screening

Theme	Representative interview quotations
Perceived susceptibility	
Robustness	I am healthy and do not think that I will have cancer.
Anxiety	I might have cancer someday although I do not express this feeling. Therefore, I purchased private cancer insurance for myself.
Perceived severity	
Death sentence	I would think that I had received a death sentence. I would spend all of my money and would think I was going to die if I had cancer.
Perceived benefits	
Early detection and treatment	Screening would help me detect cancer and get early treatment if I had cancer.
Relief or safety	I was relieved after I underwent cancer screening and heard I did not have cancer.
Taking advantage of the medical payment support program from the government	If I had cancer, I would get financial help from the National Health Insurance because I had cancer screening as requested by the National Cancer Screening program.
Perceived barriers	
No symptoms	I did not have cancer screening because I was not sick.
Self-care when having symptoms	My friend had stomach pains and self-diagnosed and self-treated by taking antacids such as Galpos. I told them to go to the hospital for screening, and they were diagnosed with stomach cancer.
Distrust of tests, doctors, and hospitals	Cancer screening tests, especially conducted by the National Cancer Screening program, are too simple and perfunctory and have made many misdiagnoses. Therefore, I do not trust results of cancer screening tests.
	I do not trust small clinics or hospitals in regional areas because they are lower in quality than large hospitals in big cities. Large hospitals have the newest medical equipment, and doctors at big hospitals have much more experience from seeing many cancer patients.
Unkind health care providers	From my experiences with cancer screening, I do not like the coldness in the nurses’ voices. Health care providers at hospitals who provide services were unkind and bothered me.
Financial issues	The national cancer screening program provides us with inexpensive or free basic cancer screening tests, but I have to pay more money for in-depth medical examinations, which is a burden for me.
Discomfort during cancer screening	I do not like being naked in bed for the pap-smear test and drinking water-like medicine for colonoscopies. Mammograms hurt my breasts, so I do not like to have breast cancer screening.
Self-efficacy	
Confidence	I can get cancer screening if I want to. I received a call from a clinic and was asked to get cancer screening. I went to the clinic and the health care providers told me what to do to get cancer screening at the clinic or a hospital.
Knowledge of cancer causes	
Family history	Cancer has a heredity basis
Food	Burnt food causes cancer.
	Food that is too salty and spicy causes cancer.
Infection	If you do not sleep with a man, you do not have cervical cancer.
Stress	Lots of stress causes cancer.
Personality	An introvert who does not get angry and makes sacrifices would be more likely to get cancer.
Body overuse	People who use their shoulders and hands a lot, such as hairdressers or butchers, are more likely to get breast cancer.

When severity of cancer was discussed among the participants, almost all of them were afraid of cancer because they would face physical pain, mental distress, a financial crisis, and social isolation. An older man said, “Cancer causes physical pain and emotional stress, so I would feel like my whole world had crashed down on me.” A majority of the participants thought that cancer would put them in financial crisis, and they would face death. Some participants acknowledged that the severity of cancer might be different at different stages. An early-stage cancer might be treatable, and a person’s condition would not be serious, but late-stage cancer would be very serious because it is often terminal.

Participants understood that the benefits of cancer screening are early detection and treatment, a feeling of relief or safety after screening, and taking advantage of the Medical Payment Support program offered by the Korean government. They believed that cancer screening for early detection and treatment of cancer could help them live long and healthy lives. Another benefit of cancer screening was relief when a health care provider gives them the results: If they were healthy and without cancer, they would feel safe, or if they had cancer, they would be glad that the cancer was detected early and could be treated.

Many participants had had cancer screening because they wanted to take advantage of the Medical Payment Support program. This program [29] provides some medical expenses to low-income Koreans with cancer, but it only benefits people who participate in the National Cancer Screening program [29]. Thus, many have had cancer screening because of this system. An older man said,

The National Health Insurance Service told me to have cancer screening every two years. Therefore, I did. If they had not told me this, I would not have done it. If I did not do it, I would have paid a penalty to the national health care system. Additionally, if I did not have cancer screening conducted by the National Health Insurance Service and I later found that I had cancer, I would have had to pay all the medical treatment expenses without any financial support from the Service. I got a call from them, and they gave me of all this information.

Six themes emerged from the many barriers to cancer screening that focus group participants identified: (a) no symptoms, (b) self-care when having symptoms, (c) mistrust in tests, doctors, and hospitals, (d) unkind health care providers, (e) financial issues surrounding cancer screening, and (f) discomfort during cancer screening. The participants, especially elderly ones, revealed that they would seek medical care only if they had symptoms. They said there was no need to get cancer screening if they had no symptoms. Many participants also said that when they had symptoms, they self-diagnosed and self-treated their abnormal health conditions. When they got sick, then they went to the pharmacy without health screening tests and asked for generic drugs to treat their symptoms. Serious consequences such as late diagnosis of cancer or death could result from these delays.

Surprisingly, participants revealed widespread mistrust in tests, doctors, and hospitals. The National Health Insurance Service provides a cancer screening program to Koreans, but participants had the impression that when they were screened, many misdiagnoses were made in the test results and their interpretation. Participants also mentioned that they had heard of many cases where doctors misdiagnosed patients. Many participants had experienced erroneous test results or misdiagnoses at private clinics and small hospitals. They preferred to get cancer screenings at large general hospitals in big cities in Korea such as Seoul, with more advanced and newer medical technology and experienced doctors. Some participants noted that they did not trust test results from one hospital and therefore went to a second hospital to get the same tests. One participant said,

Cancer screening tests conducted by the National Health Insurance Service were cheap or free of charge but, as far as trusting the tests, I wondered if I would get the correct test results. The National Health Insurance Service did not offer a variety of screening tests, only the basic ones.

I heard people around were misdiagnosed with the doctors saying they had cancer when they did not, or the doctors not finding cancer although they did have it. My wife recently was screened at a regional hospital and the doctor said that she had to get surgery and chemotherapy because she had late-stage lung cancer. Then, we went to a large-sized hospital in Seoul and she was tested again, and the doctor said it was not lung cancer and instead my wife had heart trouble. We were at a loss for words.

Regarding other perceived barriers to cancer screening, unkind health care providers including busy doctors or nurses who did not explain the test procedures and results in detail discouraged the participants from having the tests. Participants wanted to have advanced cancer screening tests that could provide more detailed information about their health in addition to the specific cancer ones provided. However, more tests cost money, and they felt that this would be a financial burden. Unpleasant and uncomfortable procedures were another barrier to cancer screening.

Regarding self-efficacy, almost all of the participants were confident that they could have cancer screening if they wanted to. The National Health Insurance Service advised them when and where they could go for screening. When they went a clinic or hospital for screening, nurses and other health care providers told them what to do.

During the focus group interviews, participants shared their knowledge of cancer causes, described in Table 3. Participants mentioned family history, unhealthy food consumption such as burnt foods, infection, and stress as causing cancer. A few mentioned introspective personality and body overuse as specific causes. Complex causes of cancer were mentioned by participants including the following:

People who get stressed and have cancer genes that were inherited from their parents will get cancer. I think 30–40% of cancer cases are attributed to stress and family history.

Most Koreans think that, if people get stressed, the blood becomes cloudy and they get cancer.

People who eat salty and spicy food, are extremely sensitive, have cancer genes, and are under stress will get cancer. Among those causes, stress is the most important.

Women who do not sleep with men do not get cervical cancer.

Introverts who put things in their minds and suppress their anger will get cancer because they do not relieve their stress.

Additionally, some participants believed that overuse of their body caused cancer:

Jobs that require shoulder and hand use cause breast cancer, and mothers who breast feed many times do not get breast cancer.

### Preferred strategies for cancer screening

Participants suggested various cancer screening strategies based on their previous experiences or preferences (Table 4). The most important preferred strategy given by most participants was group interventions by their family, friends, or acquaintances. Participants said that if someone were willing to go to the hospital with them, they would definitely have cancer screening tests. The participants said that, for them, effective information delivery methods to persuade them to have cancer screening would include: documentary videos, booklets with large font sizes, messages on smartphones, tailored letters concerning their health and personal status (e.g., family history, disease, or age) and cancer screening education meetings and seminars at churches, community centers, or senior centers. They stated that important information to be distributed should include the following: (a) people should go for cancer screening when they have no symptoms, (b) information to restore their trust in cancer screening tests, doctors, and hospitals, (c) information about hospitals and their specialties, and (d) an emphasis on a positive outlook for both healthy people and cancer patients. They mentioned that there is a great need to develop systems to provide convenient and free or low-cost services and more information on cancer screening tests and special hospitals so that they could easily go for cancer screening at their convenience. Also, kind health care providers who would explain their health conditions in more detail would encourage them to have cancer screenings.

**Table 4:** Preferred strategies for cancer screening

Theme	Representative interview quotations
Group interventions with family, friends, or acquaintances	I would be encouraged and go for cancer screening if my children helped me. If friends or acquaintances recommended that I go for cancer screening, I would go with them.
Various delivery methods	I would like to watch a video or documentary. I would like to read booklets with a large-size font. I prefer cancer education at church or a senior center. Smart phones would be good for cancer programs. I prefer tailored letters for my status including information on family history, disease, age, etc.
Information content emphasizing importance of cancer prevention	I would like a cancer program to inform me that, despite having no symptoms, I need to have cancer screening.
Convenient, free, or inexpensive services	There is a need to develop a system for people to have various inexpensive or free cancer screening tests and to use them with ease.
Kind health care providers	Health care providers need to provide really good services to people, as if they were family, and explain my health status in detail.

### Differences in beliefs, knowledge, and preferred strategies by age, gender, and residency

We did not find differences by age, gender, or residency in themes about beliefs, knowledge, or preferred strategies except for different concerns by age group. Regarding age differences in perceptions of what would happen if they got cancer, middle-aged participants were concerned about their social inability to play the role of parent and spouse in the family and of employee at work, while older participants worried about physical pain and discomfort. A middle-aged woman said,

I would worry about not playing a role in taking care of my children and spouse as well as being concerned about myself if I had cancer.

On the other hand, an older woman said,

I would be afraid of the pain, not death, if I had cancer.

## Discussion

This study provides new insights into beliefs and knowledge about cancer screening as well as preferred strategies for increasing cancer screening from the participants’ perspectives. Viewed through an HBM framework, they ranked high on their perceptions of the severity of cancer (i.e., they believed cancer causes serious consequences), they had sufficient self-efficacy (i.e., they were confident in their ability to obtain cancer screening), and they knew the benefits of screening. However, most of them ranked low on their perceived susceptibility to cancer (i.e., they did not consider themselves susceptible to cancer), felt that there were many barriers to cancer screening, and had some incorrect knowledge of the causes of cancer.

Regarding perceived susceptibility to cancer, the participants seemed to be optimistic and minimized their chances of getting cancer. The participants believed in the Korean proverb, “words have a way of coming true,” and they wanted to believe that they were healthy. Even though they were anxious about their health, they did not express their anxiety about cancer. A similar tendency to think, “I am okay, I will not get cancer” was reported in a study of Korean Americans [30], which suggests that optimism about health appears to be a Korean cultural belief that could affect cancer screening behavior. Other Asians seem to have a similar belief about words have a way of coming true. In a study [31], one immigrant Chinese cancer survivor in Australia said that “I might not have cancer if you [Doctor] didn’t mention it,” indicating misunderstanding about cancer curse etiology. This cultural belief (i.e., what people say will happen) may explain the tendency to believe in one’s own health, not to express anxiety about cancer, and not to even mention cancer.

Many of the participants thought that preventive cancer screening was not necessary as long as they were asymptomatic and felt well in general. This study confirmed that many Koreans have an orientation toward crisis health management (i.e., they see a doctor only when they are sick), similar to Korean Americans [30] and Hispanics [32]. Moreover, they performed self-care including self-diagnosis and self-treatment when they had symptoms. A previous study [33] reported similar findings on self-care for health in Koreans using healthy foods, exercise, peaceful minds, or herbal medicine. Historically, Koreans were likely to seek help from multiple sources including folkways and religion as well as modern medicine [33]. A crisis health orientation and self-care may result in serious consequences such as late diagnosis of cancer or death. In fact, colorectal cancer is diagnosed late in Koreans. According to the Korean Society of Coloproctology, of 100,895 Koreans who were diagnosed with CRC after having colonoscopies, 51.6% had stage 3 or 4 colorectal cancer and only 19.9% had stage 1 and 28.5% had stage 2 [34].

Surprisingly, participants showed widespread mistrust in cancer screening tests, doctors, and hospitals. This mistrust included beliefs that (a) cancer screening tests, especially those conducted by the National Cancer Screening program, were too simple and perfunctory and reported incorrect test results, (b) doctors made misdiagnoses, and (c) clinics and small hospitals provided a low quality of care. Previous personal experiences, the experiences of family members and acquaintances, and sensational cases from TV or in the newspapers influenced this mistrust in health care. Several participants did not trust the health care system because they knew individuals who had had negative results on cancer screening tests and died of cancer a few months later or who were diagnosed with terminal cancer and survived for years without medical treatment. Mistrust has been identified as a barrier to cancer screening in other studies of Koreans [4, 16]. A survey of 4131 Korean cancer-free men and women found distrust of screening as a barrier to using the National Cancer Screening program over an individual’s lifetime [4]. Additionally, a study using focus group interviews with 23 Medical Aid program recipients reported that the most common reason for not having cancer screening tests was a lack of trust in the National Cancer Screening program and in cancer screening units due to a low quality of service [16]. Findings from our study show that both Medical Aid program recipients and National Health Insurance beneficiaries had widespread mistrust in cancer screening tests, health care providers, and health care organizations.

Koreans’ concerns about misdiagnosis are significant. In fact, the Korea Consumer Agency [35], a governmental organization to protect consumer rights, reported that cancer misdiagnosis rates (296 cases) accounted for 61.7% of all misdiagnoses (480 cases) from 2012 to 2015. These cases included cancer that was not detected in people who had it or treatment such as surgery that was given to someone who did not have it [35]. Lung cancer (20.3%) was the most misdiagnosed, followed by breast cancer (16.2%), gastric cancer (13.2%), liver cancer (12.2%), and colorectal cancer (8.4%) [35]. Cancer was the most misdiagnosed at small, secondary hospitals (38.5%), followed by primary clinics (37.2%) and tertiary general hospitals (24.3%) [35].

Mistrust in tests and doctors may be related to test sensitivity (the ability of the test to identify correctly those who have the disease) and specificity (the ability of the test to identify correctly those who do not have the disease). For example, the Pap test has a sensitivity of 51% and specificity of 66.6%, with a higher rate of false-positive results in young women [36]. Because the Pap test does not identify cancer correctly 100% of the time, Koreans do not seem to trust it. Given the fact that it is difficult to find a test that has both high sensitivity and specificity, a clear explanation of this limitation should be provided. Furthermore, because people have misperceptions about screening, it is important for health care providers to give them accurate information about screening procedures and results in order to restore their trust in cancer screening tests.

Trust is critical to patients’ willingness to seek care and follow physicians’ recommendations [37]. Although some studies have found that physician recommendation is a powerful factor in influencing cancer screening, physician recommendations may not influence cancer screening utilization in Koreans [38, 39]. In addition, Koreans’ mistrust of clinics and small hospitals sends thousands of Koreans to large-sized general hospitals for medical tests or treatment. According to the National Health Insurance Service, the number of clients at the five largest general hospitals in Seoul increased from 19,791 in 2010 to 25,109 in 2015 [40]. This accounts for 7–8% of all clients in all health care institutions in Korea; it also accounts for 35–37% of the tertiary general hospital patients in Korea [40]. So, a relatively small number of the largest hospitals in Korea provide care for about one third of its patients. In general, lack of understanding of, or familiarity with, the health care system contributes to mistrust of health care [41]. Small hospitals with limited facilities diagnose diseases provisionally, not definitively, and recommend that people have further screening tests at larger hospitals, which may cause people to have negative impressions of small hospitals. This could be solved by the health care providers who provide screening procedures communicating to patients that further in-depth medical examinations will help the providers make more accurate diagnoses.

Participants in this study had some knowledge of the causes of cancer, but this knowledge was occasionally insufficient and incorrect. Although family history, unhealthy food consumption such as burnt food, and infections were well-known to cause cancer [42], the participants mentioned that psychosocial factors including stress and personality also caused cancer. However, studies have so far indicated that personality and perceived stress are unlikely to be related to cancer, although stress can change the way the immune system functions [43–46].

The belief that stress and personality are significant causal factors in cancer is rooted in cultural beliefs about the mind-body relationship [47]. Historically, Koreans have been influenced by Taoism, methods for cultivating mind and body and harmonizing with nature [33], which may have influenced participants to believe in the relationships among stress, personality, and cancer. In addition, some participants believed incorrectly that the overuse of the body causes cancer. These beliefs about the causes of cancer illuminate the importance of interventions containing sufficient and accurate information.

Issues related to the health care system in Korea, including the operation of the National Cancer Screening program in the National Health Care system are unique to Korea and were frequently mentioned by participants. They perceived taking advantage of the government’s Medical Payment Support program as a benefit of cancer screening. Because of this system, the participants mentioned that they underwent cancer screening even though they felt it was unnecessary. So, although a crisis health orientation and self-care are individual barriers to health care utilization among Koreans, the Medical Payment Support program facilitates cancer screening because the program only supports persons who participate in the National Cancer Screening program. This shows that individual barriers such as a crisis health orientation and self-care can be overcome by benefits of participating in the National Cancer Screening program. On the other hand, perceived barriers to cancer screening such as mistrust of tests, doctors, and hospitals; unkind health care providers; financial issues regarding medical examinations; and discomfort during cancer screening are directly related to cancer screening utilization in the health care system. Similar findings of issues related to the health care system, including a lack of interaction between the doctor and the patients, and dissatisfaction with the service system were also found in a study of cervical cancer screening using a qualitative and exploratory design with focus group interviews with 23 women aged 27 to 37 [10]. These latter findings suggest that the Korean health care system needs to be modified to improve the quality of cancer screening tests, health care providers, and health care organizations, including test procedures and heath care services.

Few previous studies have addressed preferred cancer screening strategies that Koreans suggested as possibly successful and effective interventions. The key suggestions for increasing cancer screening were removing barriers by (a) providing group interventions; (b) changing individuals’ beliefs with content that removed cognitive barriers to cancer screening; and (c) changing the health care system to provide convenient, free, or low-fee services and kind health care providers. It was not surprising that the participants preferred group interventions, as Korean culture is based on Confucian values of collectivism emphasizing the group and its interests [30, 48–50]. It is expected that group interventions can be effective for changing Koreans’ health and health care behavior. As the participants also suggested, both individual beliefs and knowledge and components of the healthcare system should be changed to increase cancer screening rates in Koreans. Studies have shown that community-based intervention using posters, leaflets, and education [51], navigators [52], video program [53], tailored telephone counseling, and postcard reminders [54] have increased cancer screening among Koreans. These intervention studies aimed to change individual beliefs and knowledge. Our findings suggest that higher participation in cancer screening will result from changes related to the health care system (e.g., providing screening tests at low cost or utilizing health care providers who are kind to patients) in addition to individual factors (e.g., beliefs and knowledge).

There are several limitations to this study. Results from this descriptive qualitative study may differ from studies of Koreans who are young, or have a history of cancer or first-degree relatives with cancer because this study focus on Koreans who middle-aged and older and who do not have first-degree relatives with cancer. However, findings from this study could be useful for developing larger-scale quantitative surveys or interventions to increase cancer screening.

We make several recommendations for future practice and research. First, it is important to restore trust in cancer screening tests, health care providers, and health care organizations by improving the quality of the health care system. Cancer screening tests should be appropriately sensitive and specific and produce correct results and diagnoses. If necessary, additional tests should be done to confirm results. Furthermore, additional by reducing the financial burden. Clinics and hospitals need to improve their quality of cancer diagnosis and treatment by providing high-quality care from well-trained, kind health care professionals. Also, expanding the benefits of cancer screening to more people in the health care system would be helpful. Many participants had cancer screening because barriers (e.g., crisis health orientation and self-care) were outweighed by benefits (e.g., the Medical Payment Support program). Currently, the Medical Payment Support program provides some medical expenses only to low-income Koreans with cancer. If more socioeconomically diverse Koreans could receive this benefit from the government, cancer screening rates would increase. Individual beliefs and knowledge might be changed through various intervention methods. Critical and accurate information on cancer and cancer screening tests should be provided. Specifically, education should stress that even individuals who are asymptomatic need to have cancer screening, and individuals who have symptoms should not self-diagnose or self-treat and should instead go to medical facilities to avoid serious consequences, such as late diagnosis of disease. Finally, preferred strategies that the participants suggested need to be tested in research and in practice to prove their efficacy for cancer screening.

## Conclusion

Theory explains and predicts health behavior and suggests ways to change this behavior. This HBM-based research enabled us to understand Koreans’ cancer screening behaviors and suggest preferred strategies to increase cancer screening utilization in Koreans. This study suggests that we (a) pay more attention to low perceived susceptibility to cancer, many barriers to cancer screening, and incorrect knowledge, and (b) improve the quality of health care and use information-delivery interventions preferred by participants and tailored to Koreans’ cultural beliefs and knowledge to increase cancer screening rates in Korea.

## Abbreviations

HBM: Health belief model
PI: Principal investigator
US: United States
